# Spectral CT imaging for assessment of metastases in melanoma patients: multi-reader evaluation

**DOI:** 10.1186/s40644-025-00889-7

**Published:** 2025-06-16

**Authors:** Christian Nelles, Philip Rauen, Franziska Meyer, Anja Dobrostal, Pia Lena Niederau, Hasan Zaytoun, Mathilda Weisthoff, Pascale Bernard, Carola Heneweer, Thomas Dratsch, David Maintz, Jonathan Kottlors, Nicole Kreuzberg, Nils Große Hokamp, David Zopfs, Thorsten Persigehl, Simon Lennartz

**Affiliations:** 1 Institute for Diagnostic and Interventional Radiology, Faculty of Medicine, University Hospital Cologne, University of Cologne, Cologne, Germany; 2 Department of Dermatology and Venereology, Skin Cancer Center at the Center of Integrated Oncology (CIO) Köln Bonn, University Hospital of Cologne, 50937 Cologne, Germany

**Keywords:** Melanoma, Metastases, Dual-energy computed tomography, Staging

## Abstract

**Background:**

Pilot studies have indicated diagnostic benefits from using dual-energy CT (DECT) for staging and follow-up of melanoma patients. The purpose of this study was to investigate the sensitivity, specificity and qualitative assessment of spectral image reconstructions for metastases in melanoma patients in a large-scale, multi-reader evaluation.

**Methods:**

In total, 308 patients with melanoma, 95 patients with metastases and a control group of 213 patients without metastases, who underwent oncologic staging CT of the chest, abdomen and pelvis on a dual-layer dual-energy CT system (dlDECT) were retrospectively included. Conventional images (CI), color-coded iodine overlays (IO) and virtual monoenergetic images at 40 keV (VMI_40keV_) were reconstructed. 6 radiologists (3 experienced with 6 to 9 years and 3 less experienced with 2 to 4 years of experience) read all cases in a CI-based session, and a session based on a combination of CI, IO and VMI_40keV_. Readers were asked to determine presence of metastases in specific tissues in a binary fashion and to indicate diagnostic certainty and lesion delineation on 5-point Likert scales.

**Results:**

Sensitivity for detection of metastases in the skeletal muscle and peritoneum was significantly higher for the spectral assessment (for skeletal muscle 70% vs. 61%; for peritoneum 76% vs. 62%, both: *p* < 0.05). For subcutaneous metastases, there was a significant increase in specificity (92% vs. 89%, *p* < 0.05), however accompanied with a significant decrease in sensitivity (79% vs. 85%, *p* < 0.05). Diagnostic certainty was rated significantly higher for spectral images than CI in all (6/6) of the assessed tissues, whereas improvements in lesion delineation were noted for the skeletal muscle, the subcutaneous tissue and the pancreas.

**Conclusions:**

We found that in melanoma patients, the benefit of dlDECT-derived spectral reconstructions depends on the assessed tissue. While assessment of skeletal muscle and peritoneal metastases was significantly improved, low or absent iodine uptake of subcutaneous lesions led to false negatives and a consecutive decrease in sensitivity.

## Background

Malignant melanoma is a common oncological disease in the western world with a significant increase in incidence over the last decades [1, 2]. It is an aggressive malignancy which may metastasize throughout the body, including tissues which are rarely affected by metastases of other oncological diseases such as the skeletal muscle or subcutaneous fat tissue [3, 4]. In the most common staging system, developed by the American Joint Committee on Cancer (AJCC), the anatomical location of distant metastases defines the subcategories of the M-stage, which are associated with different prognoses [5]. Further, it has been shown that patient outcome is influenced by the number of metastases [6]. These findings underscore the importance of accurate radiological assessment of metastatic burden in patients with malignant melanoma.

For staging of patients with grade III or IV melanoma, computed tomography (CT) of the chest, abdomen and pelvis or FDG-PET/CT are the recommended standard imaging modalities for detection of distant metastases with the exception of brain metastases, for which contrast-enhanced MRI is recommended [7, 8]. PET/CT has been established superior to other imaging modalities for detection of distant body metastases at staging; however, because of the broader availability compared to PET/CT, in many countries, contrast-enhanced CT of the chest, abdomen and pelvis remains the most widely used imaging method in clinical routine [7–9]. Moreover, there is a lack of evidence suggesting an improved accuracy when using FDG/PET-CT for follow-up of those patients under systemic therapy [7]. Although improved by the use of iodinated contrast agents, the accuracy of CT in detecting metastases remains limited due to its inherently poor soft tissue contrast [3, 10, 11]. This particularly applies to the scenario in which the contrast between the lesion of interest and the surrounding tissue remains low despite contrast medium administration.

In recent years, dual energy computed tomography (DECT) has been shown to be a promising tool in mitigating this shortcoming and improving detection of metastases of various primary tumors [11–16]. In particular, DECT derived virtual monoenergetic images at low keV levels (VMI) and iodine overlay images (IO) have been reported to be useful in this regard by highlighting contrast-enhancing structures and thereby increasing lesion-to-tissue contrast [11–16]. Therefore, these reconstructions may be particularly suitable in delineating metastases of melanoma, which are frequently hypervascular, resulting in increased iodine contrast enhancement [17]. Results of previous small-scale studies are consistent with this hypothesis, showing an improved detection of melanoma metastases at different anatomical locations, yet especially in skeletal muscle, by VMI and IO [11, 16].

However, larger-scale studies justifying reconstructing and using those DECT images regularly for melanoma staging and follow-up are still lacking to this date. Therefore, the aim of this study was to investigate whether a combination of dual-layer dual-energy CT (dlDECT) derived VMI at 40 keV (VMI_40keV_), IO and conventional images (CI) can improve detection rate, delineation and diagnostic certainty in the assessment of different metastases compared to an assessment based on CI alone.

## Methods

### Image acquisition and post-processing

All CT examinations in this study were performed on a dlDECT system (IQon, Philips Healthcare, Best, The Netherlands). dlDECT registers lower-energetic photons within an upper layer, whereas higher-energetic photons are detected within the subjacent layer, which allows for simultaneous acquisition of low- and high-energy data sets and therefore dose-neutral, routine generation of spectral data without the necessity to enable spectral acquisition [18]. The scans were conducted in cranio-caudal direction while the patients were in a supine position during inspirational breath-hold. As per clinical standard, a body weight adapted amount of iodinated contrast material followed by a 30-mL saline chaser was applied via an antecubital vein (< 55 kg, 1 mL/kg; 55–120 kg, 100 mL; > 120 kg, 120 mL; Accupaque 350 mg/mL, GE Healthcare).

CI, IO and VMI_40keV_ were reconstructed from the same spectral dataset employing the vendor’s image postprocessing software (Intellispace Portal v12, Philips Healthcare). Images were reconstructed in the axial plane. CI were reconstructed using a hybrid-iterative reconstruction algorithm. IO were generated by fusion of gray-scaled CI and material-specific, quantitative iodine maps, in which the iodine content was color-coded according to a predefined scale. IO and VMI_40keV_ were reconstructed using a dedicated spectral reconstruction algorithm. Detailed scanning and reconstruction parameters are shown in Table 1.

**Table 1:** Scanning and reconstruction parameters for CT imaging

Parameter	
Pitch	0.671
Rotation time (s)	0.33
Collimation (mm)	64 × 0.625
Matrix	512 × 512
Tube potential (kVp)	120
Automatic exposure control	on (DoseRight index 17)
Volume CT dose index (mGy)	variable^a^
Iterative reconstruction (CI)	iDose 4, level 3, filter B
Iterative reconstruction (VMI_40keV_, IO)	Spectral, level 3, filter B
Reconstruction slice thickness (mm)	2
Reconstruction slice increment (mm)	1
Contrast media flow rate (ml/s)	3.5
Bolus tracking threshold	150 HU in the descending aorta
Contrast phase	portal venous phase
Delay of image acquisition	50 s after contrast media administration
Scan range	chest, abdomen, pelvis

^a^Depending on patient size and automatic exposure control

### Study cohort

After reviewing the study design, the institutional review board waived the need for informed patient consent and approved this retrospective single-center study. The study was conducted in accordance with the Standards for Reporting of Diagnostic Accuracy Studies (STARD) 2015 list [19]. To identify patients eligible for study inclusion, the radiological information and picture archiving and communication system was screened for patients fulfilling following criteria:

1. Patients ≥ 18 years who were diagnosed with histopathologically proven malignant melanoma and did not suffer from a secondary malignancy.
2. Received a clinically indicated contrast enhanced dlDECT with a venous phase acquisition of the chest, abdomen and pelvis between 06/01/2016 and 12/31/2020 during routine cancer follow-up.
3. Patients were assigned to either a metastatic or non-metastatic cohort:
  - For the metastatic group, metastases had to be present in one or more of the following organ systems: subcutaneous tissue, skeletal muscle, liver, pancreas, peritoneum or pleura. All metastatic sites had to be confirmed in a ground truth annotation as detailed below.
  - For the non-metastatic group, CT of the corresponding melanoma patients had to be free of tumor burden. Moreover, the lack of any metastases had to be confirmed after a period of at least 3 months at ground truth annotation as detailed below.

**Fig. 1:**
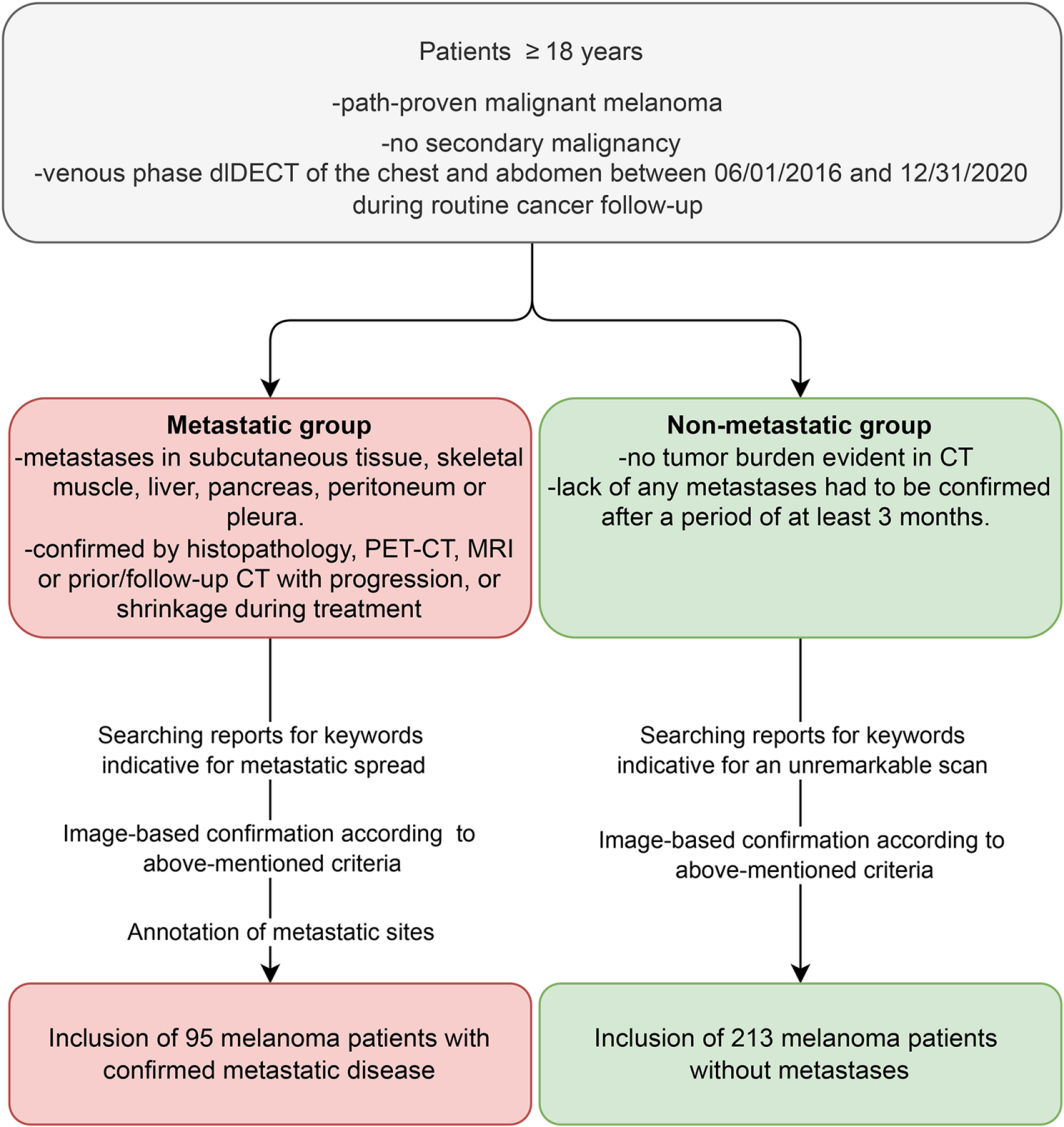
Flowchart detailing the criteria for dividing patients into two groups. Patients were selected retrospectively based on dual-layer dual-energy CT imaging data and reports from June 2016 to December 2020. The Metastatic group (red) includes patients with confirmed metastases in various organs, while the non-metastatic group (green) comprises patients with no evidence of metastases after at least three months of follow-up. The final analysis included 95 patients with metastatic disease and 213 patients without metastases. dlDECT: dual-layer dual-energy CT

The relation between metastatic and non-metastatic patients was set to approximate the chance of encountering disease recurrence in patients with advanced disease stages qualifying for cross-sectional imaging follow-up [20]. The workflow of study inclusion is displayed in Fig. 1.

### Ground truth annotation of patients with or without metastases

Assignment of patients to the metastatic or non-metastatic cohort and subsequent determination of metastatic burden was performed by a board-certified radiologist with 7 years of experience who was not involved in the further image analysis. First, radiological reports and tumor board documentation for each eligible patient were reviewed.

Patients without metastases according to those reports were confirmed as non-metastatic by reviewing follow-up imaging of at least 3 months after the scan that was included. If the follow-up imaging did confirm absence of any metastases, the patient was included as non-metastatic.

For patients with metastases, the analysis focused on the subcutaneous tissue, skeletal muscle, liver, pancreas, peritoneum and pleura. The reason for that selection was three-fold: First, the included metastases showed promising data in previous studies [11–13, 16, 21, 22]; second, some metastatic subgroups were too small to warrant adequate evaluation (e.g. spleen, kidneys or intestines); third, for some metastases, previous data led to the anticipation of diagnostic problems with spectral reconstructions (e.g., differentiation between hypervascular lymph node metastases and inflammatory lymph nodes, or problems with iodine quantification in lung nodules) [23, 24]. For patients with metastases in those tissues according to the radiological report, the corresponding CT scan were reviewed and presence or absence of metastases were annotated as binary outcomes. Lastly, metastases had to be confirmed by one or more of the following criteria:

A) Correlative imaging (i.e., unequivocal evidence of metastases in PET-CT or MRI).
B) Progression in size in prior or follow-up CT examinations.
C) Size shrinkage during anti-cancer therapy in prior or follow-up examinations.
D) Histopathological confirmation.

### Image assessment

Image analysis was independently performed by 6 radiologists, 3 of which were experienced in oncological imaging with 6 to 9 years of experience, while the other 3 were less experienced with 2 to 4 years of experience. All readers were blinded against patient group, clinical information and pre-/follow-up exams. To balance recognition bias, readers were randomly assigned to either start the assessment with the conventional reading session (including CI of all patients) or the spectral reading session (including CI, VMI_40keV_ and IO fusion images of all patients), respectively. After completing the first session, a minimum 6-week washout period was observed before the second session.

At the CI-based session, readers were presented with axial CI in the soft-tissue window. At dlDECT, CI resemble the image impression of a 120 kVp acquisition and are frequently used as clinical standard. In the spectral task, in addition to the aforementioned images, readers were presented with VMI_40keV_ as well as the color-coded IO image. Readers were asked to binarily determine the presence or absence of metastases in the following organ systems: subcutaneous tissue, skeletal muscle, liver, pancreas, peritoneum and pleura. Additionally, for each organ system, they were asked to indicate their diagnostic certainty and, in case of identified metastases, the delineation of the lesions on 5-Point Likert Scales. Likert scores are specified in Table 2.

**Table 2:** Likert scores for the qualitative criteria lesion delineation and diagnostic certainty

Rating	Lesion Delineation	Diagnostic Certainty
1	Poor delineation, barely recognizable	Very uncertain diagnosis
2	Subpar delineation, somewhat recognizable	Uncertain diagnosis
3	Average delineation, moderately recognizable	Fairly certain diagnosis
4	Good delineation, clearly recognizable	Certain diagnosis
5	Clear delineation, very well recognizable	Very certain diagnosis

### Statistical methods

All analyses were performed using software (IBM SPSS Statistics version 26.0, GraphPad Prism version 10.2.2). Sensitivity and specificity were compared between CI and spectral images using Wald χ^2^ tests. The diagnostic certainty and delineation of the lesions was compared using paired-sample *t* tests. Two-sided *p* < 0.05 was considered statistically significant. Given the exploratory nature of the research and the lack of prior effect sizes for comparison, no power analysis was performed.

## Results

### Study cohort

A total of 308 patients (mean age 62.3 ± 15.7 years), 158 men (mean age 62.0 ± 16.0 years) and 150 women (mean age 62.4 ± 15.4 years), were included in the study. The cohort with confirmed metastatic disease included 95 patients, while the cohort without metastases included 213 patients. 276 patients suffered from primary cutaneous melanoma, while there were 22 patients with melanoma of unknown primary, 2 with conjunctival melanoma, 2 with uveal melanoma and 6 with mucosal melanoma (3 sinonasal, 3 vaginal). Basic demographic information including distribution of metastases is summarized in Table 3.

**Table 3:** Patient demographics and metastatic disease distribution

Patients			
	Men		158
	Women		150
	Age		62.3 ± 15.7 years
metastatic disease	Yes		95
	No		213
metastatic siteS*	(Sub)cutaneous tissue		57
	Skeletal muscle		35
	Liver		42
	Pancreas		6
	Peritoneum		22
	Pleura		12
INitial tumor stage	**T**	**N**	**M**
0	10	50	158
1	23	98	96
2	64	54	1
3	74	49	-
4	72	-	-
x	20	12	8
Not available	45	45	45

This table details the age and gender of the patients, their status regarding metastatic disease and the specific sites affected. *some patients had metastases in multiple locations

### Image assessment

#### Lesion detection

Overall, using spectral images in addition to CI led to significant improvements in sensitivity or specificity in three of the six included tissues (skeletal muscle, peritoneum, liver), whereas no differences were observed in two tissues (pancreas, pleura) and a marked decrease in sensitivity was observed in one tissue (subcutaneous tissue).

**Fig. 2:**
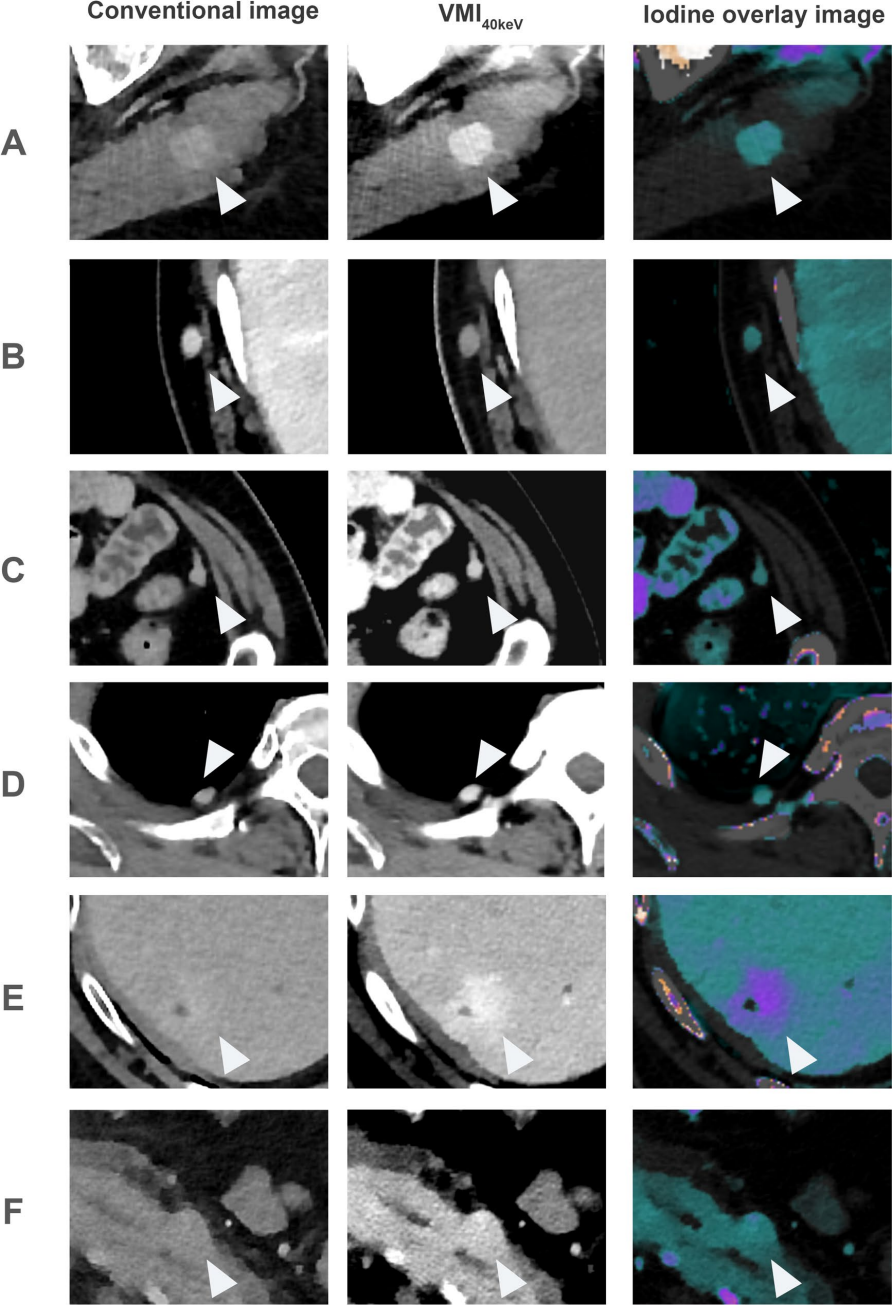
Exemplary cases of metastases in different compartments. **A**: Skeletal muscle metastasis hard to delineate in the conventional image with markedly improved delineation in virtual monoenergetic images at 40 keV (VMI_40keV_) and iodine overlays (IO). **C**: Small peritoneal metastasis adjacent to the anterior abdominal wall. **D**: Pleural metastasis with evidence of pronounced iodine uptake. **E**: Liver metastasis. **F**: Metastatic lesion in the pancreatic body more pronounced in spectral reconstructions compared to the conventional image

Across all readers, sensitivity for detection of metastases in the skeletal muscle and peritoneum was significantly higher for the spectral assessment (S) compared to the assessment solely relying on CI (for skeletal muscle S: 70% vs. CI: 61%; for peritoneum S: 76% vs. CI: 62%, both: *p* < 0.05), while there was no significant difference in specificity. For skeletal muscle metastases, using spectral images reduced the sensitivity gap between experienced and less experienced readers (CI: 66% vs. 57%; S: 71% vs. 68%). At the assessment of peritoneal metastases, readers of both experience levels improved their sensitivity, whereas this difference was statistically significant only for the subgroup of experienced readers (experienced: S: 88% vs. CI: 70%; less experienced: S: 64% vs. CI: 55%). Visualization of one exemplary metastasis per assessed tissue in CI, VMI_40keV_ and IO, respectively, is shown in Fig. 2.

**Fig. 3:**
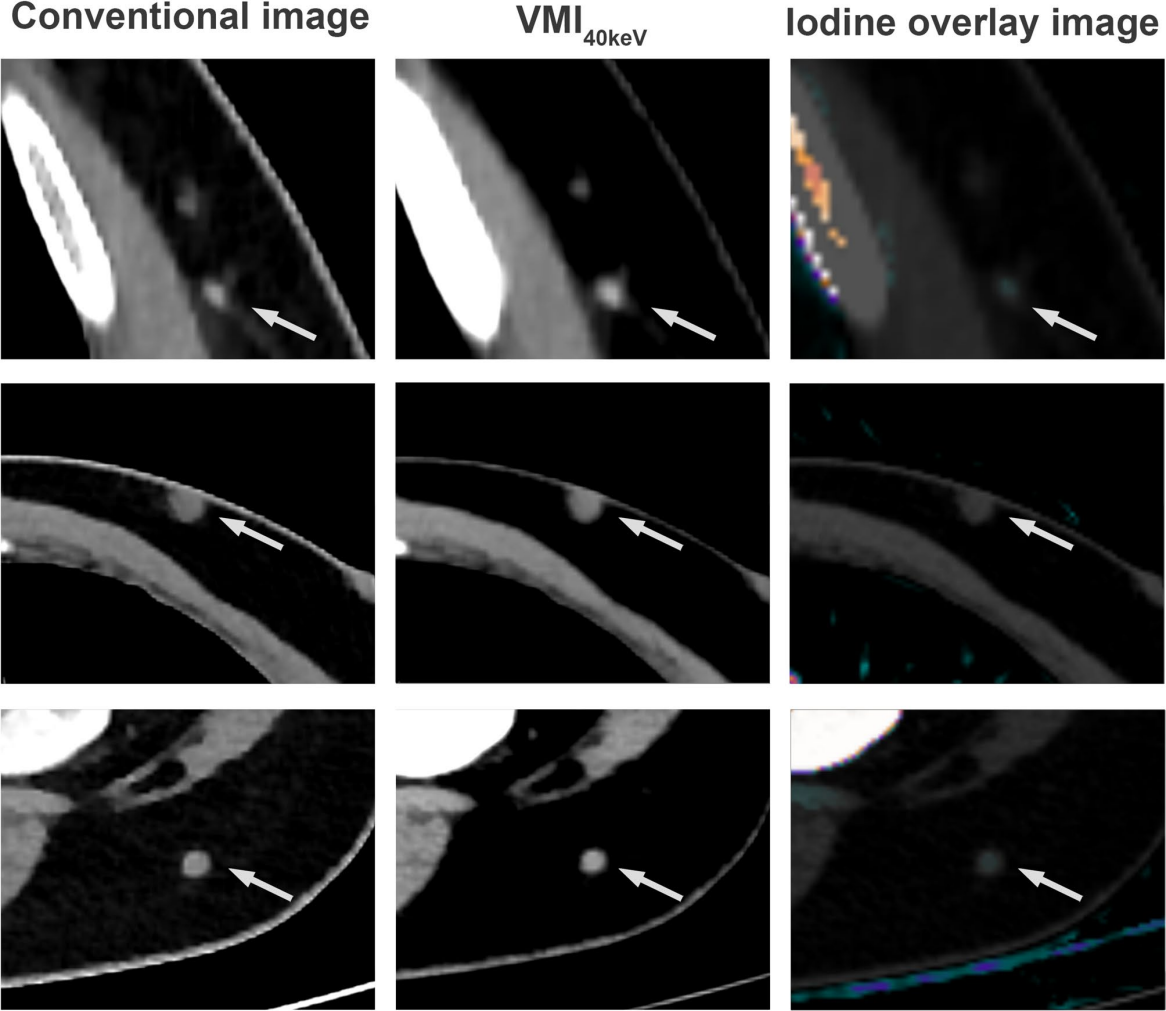
Examples of three melanoma patients with confirmed subcutaneous metastases with very low or absent iodine concentration, which may explain false-negative findings and thereby the observed decrease in sensitivity for those metastases in the assessment comprising spectral reconstructions highlighting iodine contrast (i.e., VMI_40keV_ and IO)

For subcutaneous metastases, sensitivity was significantly lower in the assessment using spectral images compared to the assessment using CI for all readers (S: 79% vs. CI: 85%, *p* < 0.05), whereas specificity was higher (all readers: S: 92% vs. CI: 89%, *p* < 0.05). Exemplary cases of subcutaneous metastases exhibiting low iodine uptake are shown in Fig. 3.

Experienced readers significantly improved their specificity in determining metastatic spread to the liver from 95 to 97% when using spectral images with similar sensitivity. For the pancreas and the pleura, no significant differences between the assessment using CI and the spectral assessment were observed.

Data on sensitivity and specificity is summarized in Table 4.

**Table 4:** Results on sensitivity and specificity for metastatic spread specified by reader experience level

		Experienced readers (*n* = 3)		Less experienced readers (*n* = 3)		All readers (*n* = 6)	
		Sens. (%)	Spec. (%)	Sens. (%)	Spec. (%)	Sens. (%)	Spec. (%)
Subcutaneous	CI	87 (149/171)*	89 (634/714)*	84 (143/171)	89 (633/714)	85 (292/342)*	89 (1267/1428)*
	S	80 (137/171)*	93 (664/714)*	77 (132/171)	91 (647/714)	79 (269/342)*	92 (1311/1428)*
Muscle	CI	66 (69/105)	98 (790/806)	57 (60/105)*	96 (778/807)*	61 (129/210)*	97 (1568/1613)
	S	71 (75/105)	98 (793/807)	68 (71/105)*	95 (763/807)*	70 (146/210)*	96 (1556/1614)
Liver	CI	90 (114/126)	95 (740/780)*	83 (105/126)	93 (727/779)	87 (219/252)	94 (1467/1559)
	S	90 (114/126)	97 (755/780)*	82 (103/126)	94 (729/779)	86 (217/252)	95 (1484/1559)
Pancreas	CI	56 (10/18)	99 (887/900)	56 (10/18)	99 (889/898)	56 (20/36)	99 (1776/1798)
	S	61 (11/18)	99 (889/899)	61 (11/18)	99 (889/897)	61 (22/36)	99 (1778/1796)
Peritoneum	CI	70 (46/66)*	97 (817/846)	55 (36/66)	97 (823/845)	62 (82/132)*	97 (1640/1691)
	S	88 (58/66)*	96 (808/845)	64 (42/66)	97 (819/846)	76 (100/132)*	96 (1627/1691)
Pleura	CI	89 (32/36)	95 (838/882)	86 (31/36)	93 (817/881)	88 (63/72)	94 (1655/1763)
	S	78 (28/36)	96 (846/881)	86 (31/36)	92 (812/882)	82 (59/72)	94 (1658/1763)

Abbreviations: CI: Conventional image assessment. S: Spectral image assessment including conventional images (CI), virtual monoenergetic images at 40 keV (VMI_40keV_) as well as iodine overlay images (IO). Bold print with asterisk indicates significant differences in sensitivity and/or specificity between conventional and spectral image assessment

#### Lesion delineation and diagnostic certainty

**Fig. 4:**
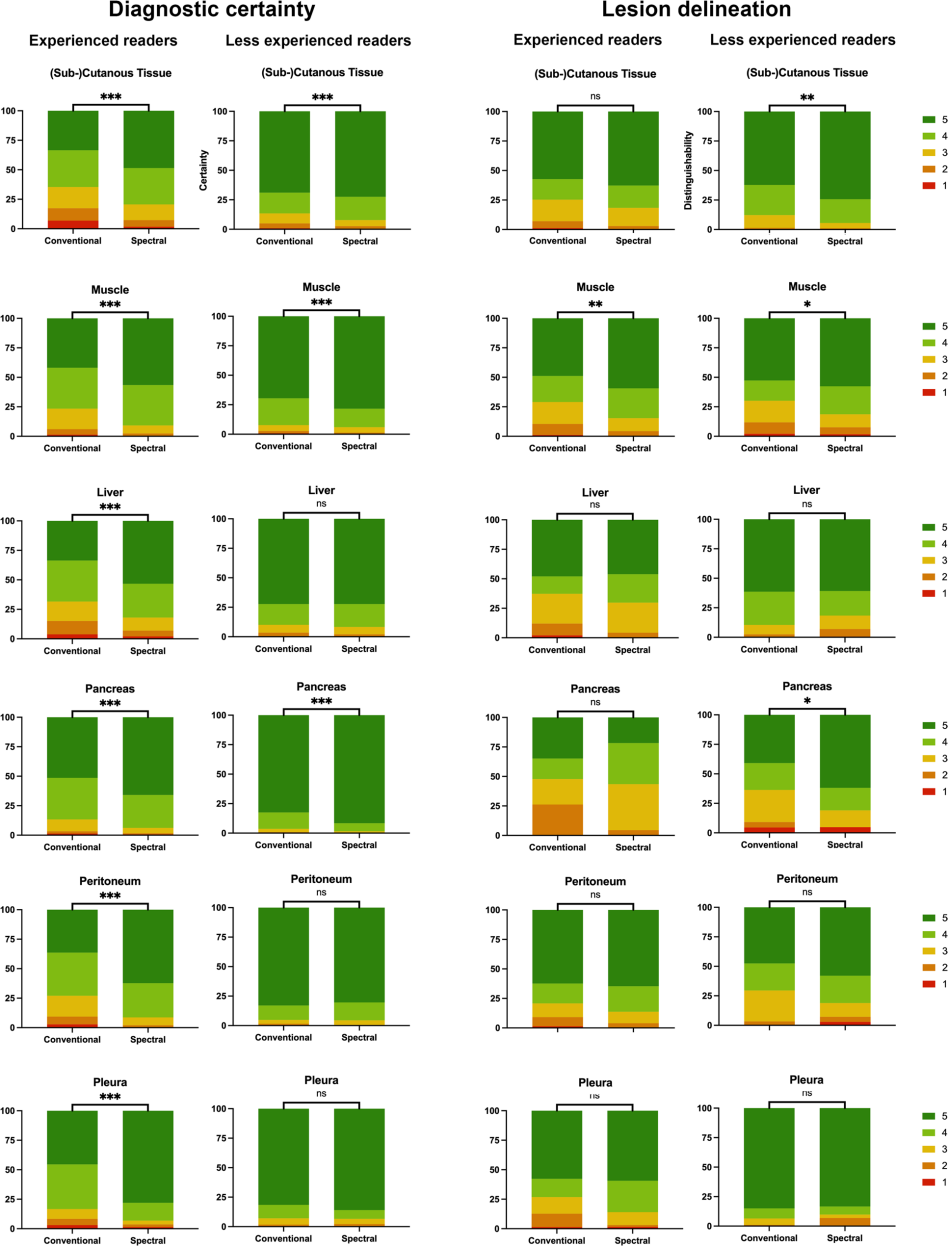
Results of the qualitative assessment regarding diagnostic certainty and lesion delineation were captured on five-point Likert scales (1: low certainty, poor delineation; 5: high certainty, clear delineation) and are presented in color-coded graphs plotted separately for each tissue type. Significantly improved diagnostic certainty at the spectral assessment was found for all assessed tissues in experienced readers. The only tissue for which spectral images could significantly improve lesion delineation for both experienced and less experienced readers was the skeletal muscle

Diagnostic certainty was rated significantly higher for spectral images than CI across all organ systems and experience levels, with the exception of the assessment of pleural metastases in the subgroup of less experienced readers, for which no significant difference was observed. Further, lesion delineation was rated higher for spectral images compared to CI for subcutaneous tissue (4.55 ± 0.76 vs. 4.36 ± 0.9) and skeletal muscle (4.34 ± 0.94 vs. 4.08 ± 1.11; all: *p* < 0.05). An overview of the average Likert scores attained for each tissue and criterion, respectively, is shown in Table 5. Detailed results of the qualitative assessment are displayed in Fig. 4.

**Table 5:** Likert scores (1–5) averaged over all readers for diagnostic certainty and lesion delineation

		Diagnostic certainty	Lesion delineation
**Subcutaneous tissue**	C	4.12 ± 1.13*	4.36 ± 0.90*
	S	4.40 ± 0.89*	4.55 ± 0.76*
**Muscle**	C	4.34 ± 0.88*	4.08 ± 1.11*
	S	4.58 ± 0.70*	4.34 ± 0.94*
**Liver**	C	4.21 ± 1.04*	4.24 ± 0.99
	S	4.44 ± 0.87*	4.24 ± 0.95
**Pancreas**	C	4.56 ± 0.74*	3.76 ± 1.10
	S	4.73 ± 0.58*	4.02 ± 1.00
**Peritoneum**	C	4.37 ± 0.93*	4.24 ± 0.99
	S	4.63 ± 0.66*	4.40 ± 0.92
**Pleura**	C	4.45 ± 0.89*	4.51 ± 0.92
	S	4.72 ± 0.71*	4.57 ± 0.86

Bold print and asterisks indicate significant differences between the assessment based on conventional images (C) and the assessment based on conventional and spectral images (S)

## Discussion

In this study, we aimed to investigate whether dlDECT derived VMI_40keV_ and IO could improve the detection of metastases in different organ systems in a large cohort of patients with malignant melanoma. For this purpose, 6 readers independently reviewed scans of the chest, abdomen and pelvis up to the proximal thighs of 308 patients, comparing a combination of VMI_40keV_, IO and CI to an assessment based on CI alone.

Recently, DECT derived VMI and IO have been shown to improve detection of metastases of different primary tumors [11–16]. However, studies that specifically investigate melanoma in this context are scarce. *Uhrig et al.* [11] investigated how many additional metastases were found by two readers in IO after reviewing CI first in a cohort of 75 patients with melanoma. In line with our results, they found an improvement of sensitivity for detection of metastases by IO, especially for muscle metastases with a marked increase from 8 to 99%. While the improvement in sensitivity regarding muscle metastases in our study was also significant, the increase was more modest (70% vs. 61%). This is most likely explained by the fact that the number of metastases included by *Uhrig et al.* was much lower. In addition to a much larger cohort, more readers with different experience levels and the evaluation of VMI in addition to IO, we used a more controlled approach by evaluating CI and spectral images in two separate rating sessions and by including patients without metastases as a control group, which reflects the clinical reality more closely.

In another previous study [16], an improvement in sensitivity for detecting skeletal muscle metastases of different primary tumors by IO and VMI was demonstrated. While the study design was similar to our current investigation, the cohort was smaller and included only 40 patients. On the other hand, analysis was conducted at a lesion level and separately for IO and VMI, while in our current study, analysis was performed somewhat broader at an organ level as well as for IO and VMI combined. The reason for this approach was that both IO and VMI have previously been shown to improve the assessment of low-contrast structures in CT and we intended to combine the enhanced visualization of iodine-enhancing structures provided by IO with the preserved grayscale image impression of VMI.

Notably, the increase in sensitivity for detecting skeletal muscle metastases using IO and VMI_40keV_ compared to CI alone was significant in the subgroup of less experienced readers (68% vs. 57%), whereas sensitivity among experienced readers remained comparable. This could be explained by the fact that skeletal muscle metastases often have poor soft tissue contrast in CI [3, 9, 10], so that less experienced radiologists are more likely to overlook them. The fact that the sensitivity gap between experienced and less experienced readers narrowed with the use of spectral images indicates that using those images may be helpful to improve diagnostic accuracy, particularly for radiologists in training.

We showed that sensitivity for detection of peritoneal metastases was also significantly higher using VMI_40keV_, IO and CI compared to CI alone, with comparable specificity. A prior study [13] investigated whether differentiation between peritoneal metastases and benign peritoneal lesions could be improved by a combination of IO and CI compared to CI alone. In this investigation, sensitivity was comparable between spectral and conventional images, while an improvement of specificity was shown (86% vs. 78%). In comparison, in our current study, specificity was already high without the use of spectral images (97%) and also without significant improvement by the additional use of VMI_40keV_ and IO. This difference could be explained by the respective control group: in the prior study, patients with benign peritoneal lesions, primarily caused by abdominal surgery, were used as a control group, while in our current study, randomly selected melanoma patients without metastases and, in most cases, without history of abdominal surgery, were used. Therefore, the rate of peritoneal abnormalities of any kind can be expected to be lower, facilitating the exclusion of peritoneal metastases and increasing specificity even without the use of spectral reconstructions.

A similar reason might explain the fact that we could not reproduce improvements in sensitivity and specificity for diagnosing pleural carcinomatosis as reported in a previous study [12]. In that study, readers were shown image sections of either benign or malignant pleural lesions and were asked to differentiate between metastatic and benign findings, taking quantitative iodine measurements into account. In terms of actual detection of the lesions, our results suggest that CI are sufficient (which is explainable due to the high contrast to adjacent lung parenchyma), whereas in case of equivocal pleural lesions, spectral images may facilitate the characterization as described before.

In contrast to skeletal muscle and peritoneal metastases, sensitivity was decreased for subcutaneous metastases by the combination of VMI_40keV_, IO and CI compared to CI alone, in particular for the experienced readers. When analyzing the respective false negative cases, we found that a large proportion of them included confirmed subcutaneous soft tissue metastases, which however did show only very subtle or completely absent iodine uptake. This explains the finding of decreased sensitivity, as the absence or low level of iodine enhancement in spectral reconstructions misled readers into classifying lesions as benign, even though these were correctly identified as metastatic based on the image impression in CI.

To our knowledge, our study represents the first larger-scale study assessing the utility of VMI and IO to improve detection of metastases of malignant melanoma in different organ systems. We showed an improved sensitivity for detection of skeletal muscle and peritoneal metastases, which both are traditionally difficult to detect in CI because of poor soft tissue contrast. These findings support a potential benefit of DECT derived VMI_40keV_ and IO for detection of metastases of malignant melanoma in these locations in routine clinical examinations. On the other hand, the decreased sensitivity for detection of subcutaneous metastases shows that there is a risk of overreliance on these spectral reconstructions. We therefore advocate for the supplementary use of VMI and IO in regular staging and follow-up of melanoma with a clear focus on assessment of the peritoneum and the skeletal muscle, considering the potential limitations that were identified in our analysis for the assessment of other tissues. Follow-up studies should focus on scrutinizing our results prospectively, analyzing diagnostic benefits of spectral reconstructions at daily clinical application.

Apart from the inherent limitations of a retrospective monocenter study design, there are other potential limitations to our study which need to be addressed: First, our study included one particular DECT platform, and although our approach was a qualitative one, inter-scanner differences regarding spectral reconstructions [25, 26] can still be considered to be a potential factor that limits generalizability to other DECT platforms. Second, although a washout period of 6 weeks was set between the spectral and CI reading sessions and the order was randomized, residual recognition bias may have remained. Yet, we aimed to balance this residual bias by letting 50% of the readers start with CI, whereas the other 50% started with the spectral image assessment. Third, it would have been preferable to use histopathological correlation for confirmation of all metastases. However, given the rather large sample size and high number of metastases per patient on average, this would have been unfeasible to achieve. Fourth, we limited the assessment to certain tissues for which assessing spectral images was deemed promising, whereas others were excluded. For instance, overlap between hypervascular lymph node metastases from melanoma and reactive/inflammatory nodes was anticipated as a potential issue based on previous data [24]; lung metastases are known to be subject to limited iodine quantification accuracy [23], particularly when being of small size; and for some metastases (e.g., spleen, renal or intestinal metastases), the corresponding subgroups in our cohort were too small to warrant adequate evaluation. Moreover, our analysis was limited to the venous phase, as our institution only includes arterial liver scans for the initial scan, weighing potential benefits in metastasis detection against the additional radiation dose during the course of follow-up. Fifth, it could be discussed whether some false-negative subcutaneous findings were caused by their small size. However, after careful consideration, we decided against a size cutoff for lesion assessment so as not to miss out on the potential benefits of spectral reconstructions for small lesions. Lastly, some of the subgroups for certain types of metastases were relatively small, which to a degree limits generalizability of our results for those specific lesions.

## Conclusions

Our results suggest that the diagnostic benefits of dlDECT-derived spectral reconstructions depend on the assessed tissue with significant improvements particularly for the assessment of skeletal muscle and peritoneal metastases and a decreased sensitivity for subcutaneous metastases. Our findings may therefore inform more tailored use of spectral reconstructions for cancer staging and follow-up of melanoma patients.

## Data Availability

The datasets used and/or analyzed during the current study are available from the corresponding author on reasonable request.

## Abbreviations

dlDECT: dual-layer dual-energy CT
CI: Conventional images
IO: Iodine overlay images
VMI_40keV_: Virtual monoenergetic images at 40 keV
